# Mechanisms of MicroRNA Biogenesis and Stability Control in Plants

**DOI:** 10.3389/fpls.2022.844149

**Published:** 2022-03-08

**Authors:** Lu Zhang, Yu Xiang, Shengbo Chen, Min Shi, Xianda Jiang, Zhuoli He, Shuai Gao

**Affiliations:** ^1^Collaborative Innovation Center for Efficient and Green Production of Agriculture in Mountainous Areas of Zhejiang Province, College of Horticulture Science, Zhejiang Agriculture and Forestry University, Hangzhou, China; ^2^Zhejiang Provincial Key Laboratory of Bioremediation of Soil Contamination, Zhejiang Agriculture and Forestry University, Hangzhou, China

**Keywords:** miRNA biogenesis, processing, transcription, stability control, Argonaute

## Abstract

MicroRNAs (miRNAs), a class of endogenous, non-coding RNAs, which is 20–24 nucleotide long, regulate the expression of its target genes post-transcriptionally and play critical roles in plant normal growth, development, and biotic and abiotic stresses. In cells, miRNA biogenesis and stability control are important in regulating intracellular miRNA abundance. In addition, research on these two aspects has achieved fruitful results. In this review, we focus on the recent research progress in our understanding of miRNA biogenesis and their stability control in plants.

## Introduction

MicroRNAs (miRNAs), which are 20–24 nucleotide long, endogenous, non-coding RNAs, repress its target gene expression through sequence complementarity. miRNAs play an important role in all aspects of normal plant growth, development, and biotic and abiotic stress (Wu et al., 2009; Budak et al., 2015; Feng et al., 2016; Shriram et al., 2016; Li et al., 2017b; Brant and Budak, 2018; Ayubov et al., 2019; Song et al., 2019). Thus, the spatiotemporal expression of miRNAs is regulated at multiple levels to ensure the fine regulation of target genes and maintain normal life activities.

The first miRNA *lin4* was discovered in *Caenorhabditis elegans* in the early 1990s (Lee et al., 1993). Researchers focus on the importance of miRNA in regulating gene expression. *MIRNA (MIR)* genes are transcribed by DNA-dependent RNA Polymerase II (Pol II), and the primary transcripts of *MIRs* (known as pri-miRNAs) are 5′ capped, 3′ polyadenylated, and/or spliced, similar to message RNA (mRNA) (Xie et al., 2005; Rogers and Chen, 2013; Hunt, 2014; Ramanathan et al., 2016; Deng and Cao, 2017).

**FIGURE 1:**
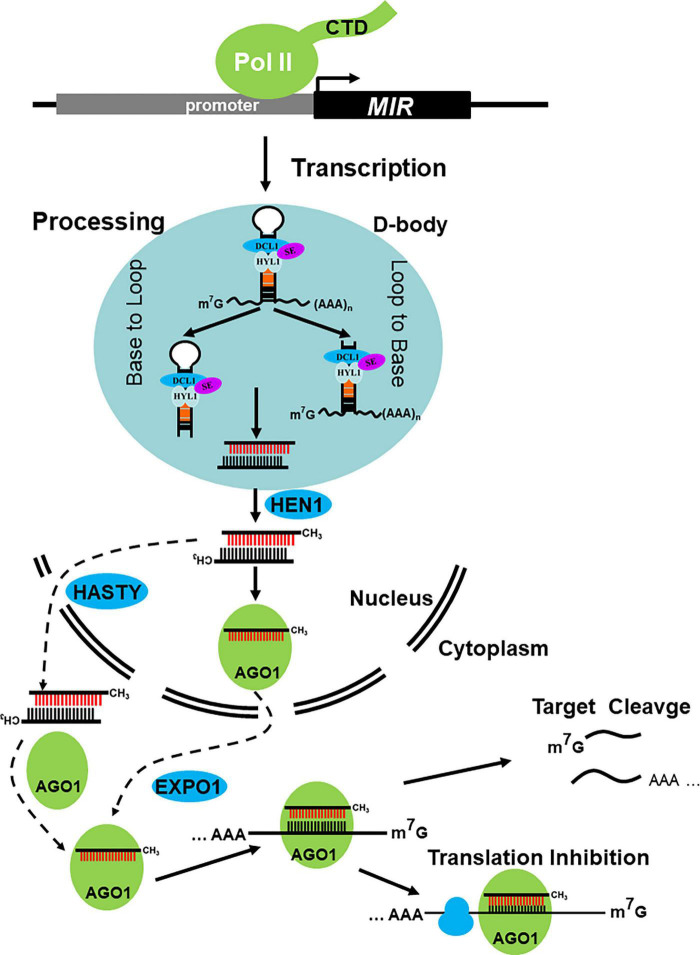
Major steps in miRNA biogenesis and action in plants. *MIR* genes are transcripted by DNA dependent RNA Polymerase II (Pol II), with that the primary transcripts (also knowed as pri-miRNAs) are processed to the miRNA/miRNA*duplex by dicing complex in D-bodies, neither from Base to Loop nor from Loop to Base. The resulting miRNA/miRNA* duplex is then methylated by terminal methyltransferase HEN1, which happens in nucleus. And then, the miRNA load into AGO1 protein and export via EXPO1 or the methylated duplex translocated from nucleus to cytoplasm via HASTY, here load into AGO. Both to form RISC to direct target cleavge and/or translational inhibition.

Pri-miRNAs, a typical hairpin-like structure RNAs, can be recognized by the processing complex, subsequently undergoing two major nuclear processing steps mediated by Dicer-like protein DCLs and two important cofactors, Serrate (SE) and Hyponastic leaves 1 (HYL1). Dicer-like 1 (DCL1) is primarily responsible for miRNA production. Notably, the production of miR839 and miR822 is dependent on DCL4 but not DCL1 (Rajagopalan et al., 2006). After the two processes, the miRNA/miRNA* duplex, which contends 2 nt overhang at its 3′ end, is released either from Loop to Base or Base to Loop (Kurihara et al., 2006; Addo-Quaye et al., 2009; Bologna et al., 2009, 2013; Song et al., 2010; Figure 1).

The miRNA/miRNA* duplex is methylated by the Hua Enhancer 1 (HEN1), a terminal methyltransferase, which methylates miRNA/miRNA* and siRNA/siRNA* duplexes on its 2′ OH of the 3′-terminal nucleotide particularly in plants (Yu et al., 2005; Yang Z. et al., 2006; Baranauskė et al., 2015). Then, the miRNA/miRNA* duplexes are thought to be translocated from the nucleus to the cytoplasm by HASTY (HST), a homologous gene of animal Exportin 5 (EXPO5) (Park et al., 2005). Afterward, the guide strand (miRNA) is loaded into Argonaute (AGO) to form RNA-induced silencing complex (RISC), whereas the passenger strand (miRNA*) is removed and degraded (Figure 1). This process is called Argonaute loading and sorting. After the formation of RISC, mature miRNAs can search its targets by base pairing. miRNA-target base pairing strengthens with nearly perfect match in plants but relatively loosens in animals. After the research period, the mode of miRNA’s action on its targets is clear, primarily in target cleavage and/or translation inhibition (Baumberger and Baulcombe, 2005; Figure 1). In addition, some miRNAs, such as miR390, miR173, and miR828 can trigger the production of secondary siRNAs, which are called phasiRNAs and/or tasiRNAs (Peragine et al., 2004; Vazquez et al., 2004; Allen et al., 2005; Yoshikawa et al., 2005; Montgomery et al., 2008; Zhang et al., 2012; Fei et al., 2013; Deng et al., 2018). Maintenance of miRNA abundance is the prerequisite for its function, and the biogenesis and stability control are the two important coincides of miRNA abundance. After nearly 30 years of intensive research, dozens of genes involved in miRNA biogenesis and stability control have been identified, and the pathway has been gradually revealed. In this review, we focus on the recent research progress in our understanding of miRNA biogenesis and their stability control in plants and pay more attention to the regulation of genes that affect the abundance of miRNA.

## MicroRNA Biogenesis

### Regulation of *MIRNA* Gene Transcription

**FIGURE 2:**
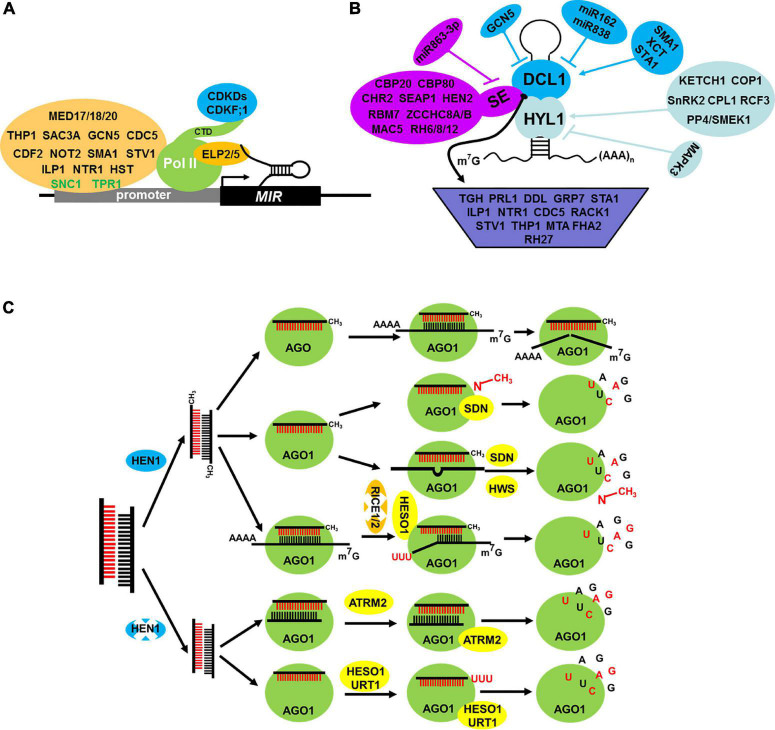
Factors involved in the regulation of MIR transcription **(A)**, pri-miRNA processing **(B)** and miRNA stability control **(C)**. **(A)** Factor in regulating MIR transcription. Several factors interact with MIR promoter and Pol II can active (orange with black text) or repress (orange with green text) the MIR transcription. Moreover, Pol II activity is subjected to phosphorylation at its C-terminal domain (CTD) via CDKDs and CDKF; 1 (blue). Elongator factor 2/5 can interact with pri-miRNAs and Pol II in transcription elongation stage to promote transcription. **(B)** Factors in regulating core processing machinery component DCL1 (blue), HYL1 (aqua), and SE (purple) and also factors that influence pri-miRNA structure, stability, splicing, loading to the processor and the processor activity (indigo blue). **(C)** Factors in regulating miRNA stability control. The AGO1 protein can protect miRNA degradation and also recruit some degradation factors to the RISC complex. HESO1 and URT1 catalyze the 3′ terminal uridylation of unmethylated miRNA and trigger their degradation, but ATRM2 degradate unmethylated miRNA/miRNA* duplex. Additionally, SDN1 degradate methylated nucleotide of 3′end of miRNA, but some factors also degradate uridilated cleavage products and also unoptimizable RISCs (RICE1/2 and HWS, respectively), all of these aspects affect the stability of miRNA and its abundance.

Similar to the formation of message RNA (mRNA), the formation of pri-miRNAs follows the same processes including transcription, capping, 3′ polyadenylation, and splicing. *MIR* genes are transcribed by DNA-dependent RNA polymerase II (Pol II). Thus, some factors associated with Pol II may also affect *MIR* transcription (Figure 2A). The mediator complex plays a critical role in recruiting Pol II to the promoters of *MIR* genes, such as MEDIATOR 20A (MED20A), MEDIATOR 17 (MED17), and MEDIATOR 18 (MED18). In these mutants, the transcription level of *pri-miRNAs* is suppressed (Chadick and Asturias, 2005; Kim et al., 2011). Tho2/Hpr2 Phenotype 1 (THP1) and Yeast Sac3 Homolog A (SAC3A), two core subunits of the Transcription Coupled Export 2 (TREX-2) complex, interact and colocalize with Pol II to promote *MIR* gene transcription (Zhang et al., 2020). The C-terminal domain (CTD) of Pol II can be phosphorylated by Cyclin-Dependent Kinase Ds (CDKDs) and Cyclin-Dependent Kinase (CDKF;1), and the phosphorylation of CTD facilitates *MIR* transcription, 5′-end capping, cotranscriptional RNA processing, and 3′-end polyadenylation (Hajheidari et al., 2012). Histone modification and chromatin remodeling are two important epigenetic modifications for gene activation and/or inhibition, and they are important for the transcription of *MIR* genes. For example, General Control Non-repressed protein 5 (GCN5), a histone acetyltransferase, targets a number of *MIR* genes, and it is responsible for the acetylation of H3K14 of these loci (Kim et al., 2009) and others, including SWINGER (SWN), CURLY LEAF (CLF), and PICKLE (PKL), which regulate *MIR* transcription as well. Different from the above, these three epigenetic factors affect only a few MIRs, for example, *MIR156A/C*, and accelerate the transition of juvenile to adult phase (Xu et al., 2016). Chromatin Remodeling factor 2 (CHR2), an ATPase subunit of the large Switch/Sucrose Non-Fermentable (SWI/SNF) chromatin-remodeling complex, positively regulates transcription of MIR loci by its chromatin remodeling activity (Wang Z. et al., 2018). Some transcription factors, such as Cell Division Cycle 5 (CDC5) and Cycling DOF Factor 2 (CDF2), also impair MIR transcription. CDC5, an MYB-related protein, positively regulates the occupancy of Pol II at *MIR* promoters and its activities (Zhang et al., 2013). CDF2, a member of DNA binding with One Finger (DOF) gene family, regulates MIR transcription positively and negatively by recruiting different transcriptional machinery to different MIR promoters (Sun et al., 2015). Apart from transcription factors, other regulators play an important role in MIR transcription, including Negative On TATA less2 (NOT2), ELONGATOR PROTEIN 2/5 (ELP2/5), Suppressor of *npr1-1* Constitutive 1 (SNC1), Topless Related 1 (TPR1), SMALL1 (SMA1), Short Valve 1 (STV1), Increased Level of Polyploidy1-1D (ILP1), NTC-Related protein 1 (NTR1), and HASTY (HST). NOT2a/2b, a pair of NOT2_3_5 domain-containing proteins, promotes the transcription of MIR genes via interacting with Pol II (Wang et al., 2013). ELP2/5, which are two elongator proteins, and loss functions of ELP2/5 reduce Pol II occupancy at miRNA loci and pri-miRNA transcription (Fang et al., 2015a). SNC1, a disease-resistant gene, and its transcriptional corepressor TPR1 repress the transcription of MIR genes (Cai et al., 2018). Recent research suggests that STV1 impresses the transcription of MIR by affecting the binding of Pol II to the promoter of MIR genes and also SMA1 (Li et al., 2017c,^2018^). ILP1/NTR1, which are two conserved disassembly factors of the Intron-Lariat Spliceosome complex, positively regulate miRNA biogenesis by facilitating the transcriptional elongation of MIR genes (Wang et al., 2019a). HST, which is the ortholog of EXPORTIN5, is considered as an exportin of plant miRNAs from the nucleus to the cytoplasm for the last two decades. However, at present, Cambiagno et al. (2021) have suggested that HST plays a role in promoting MIR gene transcription.

### Regulation of MicroRNA Processing

After transcription, the pri-miRNAs with a typical stem-loop structure are produced in the nucleus. Then, pri-miRNAs follow two constitutive cuts to produce pre-miRNAs and to release the miRNA/miRNA* duplex. This process is called processing, which occurs in two distinct subnuclear bodies, namely, Dicing-bodies (D-bodies), or SmD3/SmB bodies (Fang and Spector, 2007; Fujioka et al., 2007). DCL1, HYL1, and SE are the three core components of a microprocessor. DCL1, an RNAse III endoribonuclease, is responsible for the cleavage of pri-/pre-miRNAs. The knockout of *Arabidopsis* DCL1 is embryo lethal (Errampalli et al., 1991). HYL1 and SE are the two cofactors of DCL1, which interact with each other in D-bodies to ensure the accuracy and efficiency of processing (Fang and Spector, 2007; Dong et al., 2008; Liu et al., 2012; Zhu et al., 2013). Increasing studies have been conducted on DCL1, HYL1, and SE because of its importance in processing (Figure 2B).

DCL1 was first identified in pri-miRNAs and pre-miRNAs processing (Kurihara and Watanabe, 2004). Several factors that could regulate the transcription of DCL1, including GCN5, Stabilized 1 (STA1), and XAP5 Circadian Timekeeper (XCT), were identified (Figure 2B); the first one is a negative factor and the last two are positive ones (Kim et al., 2009; Ben Chaabane et al., 2013; Fang et al., 2015b). Otherwise, the abundance of DCL1 is fine-tuned by two feedback regulating levels. On the one hand, the biogenesis of miR162 is dependent on DCL1, but the increase of miR162 suppresses the abundance of DCL1mRNA by target cleavage. On the other hand, the generation of miR838 depends on the processing of DCL1 pre-mRNA, which leads to a decrease in productive mRNA processing of DCL1 (Xie et al., 2003; Rajagopalan et al., 2006). Except for the above, a recent research suggested that SMA1 not only affects MIR transcription but also plays an important role in the correct splicing of DCL1 (Li et al., 2018).

HYL1, an important chaperone of DCL1, encodes a double-stranded RNA-binding protein and plays a critical role in processing accuracy and efficiency. In *hyl1* mutant, more *pri-miRNAs* but less mature miRNAs accumulate (Vazquez et al., 2004). HYL1 protein comprises three representative domains, including two double-stranded RNA-binding domains (dsRBD1 and dsRBD2) in its N-terminal and one protein–protein interaction domain in its C-terminal. Yang et al. (2010, 2014) suggested that HYL1 binds to the double-stranded region of pri-miRNAs in dimer and the homodimer functions as a molecular anchor for correct DCL1 cleavage. In the past few years, several groups approached forward genetic screen of *hyl1* mutant for its suppressor. Several dominant DCL1 alleles were identified, and all of them can rescue the phenotypes of *hyl1* mutant defects in varying degrees (Tagami et al., 2009; Liu et al., 2012; Gao et al., 2020). Notably, apart from DCL1, Gao et al. (2020) identified new suppressors such as SOP1 (Suppressor of pas2 1), which is a novel factor of *hyl1*. Further study showed that HYL1 antagonizes nuclear exosome to protect pri-miRNAs from degradation. In recent years, considerable studies have been conducted on the function of HYL1, particularly in D-body formation, nuclear-cytoplasm translocation, and post translational modification (Figure 2B). MOS4-associated Complex 7 (MAC7), a component of the MAC complex, promotes the localization of HYL1 to D-bodies. In *mac7* mutant, the number of D-bodies is decreasing, which may decrease the miRNA level (Jia et al., 2017). In addition, considerable research has shown that HYL1 can localize not only in the nucleus but also in the cytoplasm. Karyopherin Enabling the Transport of the Cytoplasmic HYL1 (KETCH 1), a member of the importin β-family, transports HYL1 from the cytoplasm to the nucleus to take part in miRNA biogenesis (Zhang et al., 2017a). In dark conditions, HYL1 suffers from degradation in the cytoplasm, whereas in light conditions, Constitutive Photomorphogenic 1 (COP1), which is a RING-finger E3 ligase, translocates into the cytoplasm from the nucleus and protects the degradation of HYL1 by suppressing the activity of an unknown protease (Cho et al., 2014). Phosphorylation plays an important role in regulating the activity and stability of HYL1 (Figure 2B). The activity and stability of the phosphorylation of HYL1 affect the accurate processing of miRNA. Phosphorylated HYL1 is non-functional. C-terminal domain Phosphatase-Like 1 (CPL1) shares a domain with yeast and human Fcp1 phosphatases (Koiwa et al., 2002) and dephosphorylates HYL1, thereby leading to accurate processing and strand selection (Manavella et al., 2012). Similar to CPL1, a PP4/Suppressor of MEK 1 (SMEK1) complex can also dephosphorylate and stabilize HYL1 (Su et al., 2017). Some kinases, such as Mitogen-Activated Protein Kinase (MAPK3) and SNF1-related Protein Kinase 2 (SnRK2), can phosphorylate HYL1 and waken the position of HYL1 during miRNA processing (Raghuram et al., 2015; Yan et al., 2017). The regulator of CBF gene expression 3 (RCF3), a KH-domain protein, interacting with phosphatases CPL1 and CPL2, mediates the dephosphorylation of HYL1 and then increases its activity in specific tissues (Karlsson et al., 2015).

SE, another cofactor of DCL1, encodes a C_2_H_2_ zinc finger protein. *se* mutant exhibits some developmental defects similar to *hyl1* mutant. Further analysis suggested that SE plays a critical role in pri-miRNA processing (Yang L. et al., 2006). After years of studies, research on SE has made great progress (Figure 2B). The transcription level of SE is regulated by miR863-3P in a feedback loop manner (Niu et al., 2016). Apart from transcriptional regulation, post translational modification is important for SE. SnRK2 can phosphorylate HYL1 and SE *in vitro*, but the real function *in vivo* is largely unknown (Yan et al., 2017). Several SE-interacting proteins have been identified for the past few years. CHR2, a typical chromatin remodeling factor, which can interact with SE to remodel pri-miRNAs’ conformation and impair its processing (Wang Z. et al., 2018). MAC5, a component of MOS4-associated complex (MAC), interacts with SE and protects pri-miRNAs from nuclease degradation (Li et al., 2020). Recent study has found another SE-interacting protein, namely, Serrate-Associated Protein 1 (SEAP1), which positively promotes miRNA biogenesis by modulating pri-miRNA splicing, processing, and/or stability (Li M. et al., 2021). SE also interacts with the RNA helicase HEN2, RNA-binding protein RBM7, and one of the two ZCCHC8A/ZCCHC8B, which are members of the Nuclear Exosome Targeting (NEXT) complex. SE and NEXT complex promote the degradation of pre-miRNA, but unlike SE, the NEXT complex is not necessary for miRNA processing (Bajczyk et al., 2020). SE is not only involved in miRNA biogenesis, but also in regulating the expression of mRNA. First, SE promotes pre-mRNA splicing by interacting with Cap Binding Complex (CBC) (Laubinger et al., 2008). Second, SE plays an unexpected role in promoting the expression of intronless genes via promoting the association of Pol II with direct chromatin binding (Speth et al., 2018). Third, SE is also involved in the expression of TE by promoting H3K27me1 mediated by Trithorax Related Protein 5 and 6 (ATXR5/6) and by suppressing RNA silencing via RNA-dependent RNA polymerase 6 (RDR6) (Ma et al., 2018). Notably, SE also participates in phase separation. SE-mediated phase separation is necessary for the formation of D-bodies, in which accurate and efficient miRNA processing occurs (Xie et al., 2021). A recent study also reported that several RNA helicase RH6/8/12 interact with SE and promote the phase separation and the formation of D-bodies (Li Q. et al., 2021).

## Other Regulatory Factors Influencing MicroRNA Biogenesis

In addition to the regulation of miRNA biogenesis core components, other regulatory factors may affect the pri-miRNA structure, stability, splicing, loading to the processor, and processor activity (Zhang et al., 2015; Wang Z. et al., 2018; Figure 2B). These processes may impress miRNA biogenesis. The lengths of plant pri-miRNAs hairpin structure range from 49 to 900 nt, which affect pri-miRNA processing pattern and efficiency (Bologna and Voinnet, 2014). To date, no regulatory factors influencing cleavage pattern determination have been identified. The stability of pri-miRNA also affects processing. For example, PRL1, a conserved WD-40 protein, promotes pri-miRNAs accumulation by stabilizing pri-miRNAs and enhancing the DCL1 activity (Zhang et al., 2014). Similar to PRL1, DAWDLE (DDL), a forkhead domain (FHA) containing protein, may stabilize pri-miRNAs but not affect *MIR* promoter activity (Yu et al., 2008). Plant *MIRs* also suffer from splicing and alternative splicing, which may impair pri-miRNA processing, for example, glycine-rich RNA-binding protein 7 (GRP7), STA1, and ILP1/NTR1. GRP7, a hnRNP-like glycine-rich RNA-binding protein, interacts with pri-miRNAs *in vivo* and promotes pri-miRNA splicing (Köster et al., 2014). ILP1/NTR1 also influences the alternative splicing of pri-miRNAs. However, in ILP1 or NTR1 mutant, pri-miRNAs with or without introns are globally downregulated (Wang et al., 2019a). Cap Binding Protein 20 (CBP20) and 80, which are two members of the CBC complex, are involved in mRNA and pri-miRNAs splicing, but they may have a direct role in pri-miRNA processing and splicing because spliced and unspliced pri-miRNAs are increased (Laubinger et al., 2008). Apart from the core component of dicing complex HYL1 and SE, many other proteins participate during processing. They regulate DCL1 activity and/or facilitate the loading of pri-miRNAs to the processing complex by interacting with these core factors. TOUGH (TGH), a G-patch domain-containing protein, is a known member in the DCL1 complex, which positively regulates miRNA and siRNA biogenesis (Ren et al., 2012a). CDC5 plays dual roles in miRNA biogenesis, positively regulates *MIR* transcription, and/or promotes pri-miRNAs processing by interacting with DCL1, thereby increasing its activity (Zhang et al., 2013). Receptor of Activated C Kinase 1 (RACK1), a partner protein of SE, promotes miRNA biogenesis by regulating processing (Speth et al., 2013). RH27, a DEAD-box RNA helicase, which associates with pri-miRNAs and interacts with miRNA-biogenesis components, including DDL, HYL1, and SE. In *rh27-2*, a large number of miRNAs and their pri-miRNAs are suppressed in shoot apices and root tips (Hou et al., 2021). Short Valve 1 (STV1), a conserved ribosomal protein, facilitates the recruitment of pri-miRNAs to HYL1 to promote miRNA biogenesis (Li et al., 2017c). miRNA processing occurs in D-bodies; thus, its formation directly influences the miRNA level. THP1, a subunit of the TREX-2 complex, interacts with SE and facilitates the formation of D-bodies, thereby promoting miRNA processing. Some factors in mRNA modification are important for miRNA biogenesis. mRNA adenosine methylase (MTA), a homolog of METTL3, introduces N6-methyladenosine (m^6^A) into pri-miRNAs and promotes miRNA biogenesis by interacting with Pol II and TOUGH, a known regulator in miRNA processing (Bhat et al., 2020). Recently, some researchers have proposed that miRNA biogenesis may be related to light signaling. Although the abundance of DCL1, HYL1, and SE is upregulated during de-etiolation, the levels of most miRNAs are not significantly increased owing to the reduction of miRNA processing activity through an unknown suppressor and the shortening of the half-life of some miRNAs via SMALL RNA DEGRADING NUCLEASE 1 (SDN1) (Choi et al., 2019). Secondly, FHA2, a forkhead-associated domain containing protein, which suppresses miRNA biogenesis in a light-dependent manner. FHA2 promotes HYL1 binding but inhibits the binding of DCL1 to pri-miRNAs (Park et al., 2021). Therefore, these regulatory proteins in miRNA biogenesis change pri-miRNA’s structure, stability, modification, splicing state, and binding to the processing machinery, thereby influencing miRNA biogenesis.

The miRNA/miRNA* duplex is released following transcription and processing. HEN1 methylates the miRNA/miRNA* duplexes at 2′ OH of the 3′-terminal nucleotide. Then, the duplexes export from the nucleus to the cytoplasm, and the guide strand (also known as miRNA) load into the effector protein AGO to form the RISC complex. AGO1 is the major effector protein for the vast majority of miRNAs. In recent years, relevant research in AGO1 transcriptional and translational regulation (Vaucheret et al., 2006; Bortolamiol et al., 2007; Chiu et al., 2010; Csorba et al., 2010; Earley et al., 2010; Derrien et al., 2012), AGO sorting, strand selection (Mi et al., 2008; Takeda et al., 2008), RISC assembly (Iki et al., 2010, 2012; Iwasaki et al., 2010; Earley and Poethig, 2011), RISC loading, and miRISCs export (Wang et al., 2011; Cui et al., 2016; Tomassi et al., 2020; Zhang et al., 2020) have made several achievements. Here, we do not expand, and we focus on the maintenance of miRNA stability.

## MicroRNA Stability Control

Maintenance of intracellular miRNA homeostasis is important for plants in the adaption of environmental and developmental changes. Except for miRNA biogenesis, miRNA stability control also plays key roles in miRNA homeostasis regulation. Several aspects, including 3′-end modification (methylation and uridylation), endoribonuclease-mediated miRNA degradation, association with AGO protein, and miRNA-target interaction, can affect miRNA stability. The factors involved in these processes all affect the stability of miRNAs.

### Regulation of MicroRNA 3′-End Modification

A number of factors, which regulate miRNA 3′ end modification such as methylation and uridylation, have been identified (Figure 2C). First, HEN1 catalyzed miRNA/miRNA* and siRNA/siRNA* duplex methylation; although the binding of its two dsRNA-binding domains to the RNA substrate is significant, the contribution is different (Baranauskė et al., 2015). After the action of HEN1, the methyl group is added at 2′ OH of 3′ terminal nucleotide of miRNA (Yu et al., 2005; Yang Z. et al., 2006). To date, HEN1-mediated methylation is considered as the core mechanism of miRNA stability regulation. In 2017, a group from Chinese Taipei suggested that light activated the expression of HEN1. Moreover, the activation depends on a number of photoreceptors and transcription factors HY5. Further analysis found that HY5 is also targeted by miR157d, whose abundance is regulated by HEN1 (Tsai et al., 2014). This project focuses on the transcriptional and post transcriptional regulation of HEN1. Apart from methylation and nuclease media degradation of methylated miRNAs (see blow, SDNs), miRNA also suffered from uridylation. In *Arabidopsis*, HEN1 Suppressor 1 (HESO1) is the first gene identified for miRNA uridylation (Li et al., 2005; Zhao et al., 2012). HESO1 catalyzes the addition of uracils to the 3′-end of miRNAs in the absence of HEN1. Therefore, HESO1 catalyzes unmethylated miRNAs. During the overexpression of HESO1 in *hen1* mutant, the developmental defect was more serious than *hen1* mutant. Also, the miRNAs level is less, indicating that HESO1 promotes miRNA degradation (Ren et al., 2012b). The second one is UTP: RNA uridylyltransferase 1 (URT1), a functional paralog of HESO1, which functionally reduant and cooperates with HESO1 (Tu et al., 2015; Wang et al., 2015). HESO1 and URT1 colocalize and interact with AGO1 and uridylate AGO1-bound miRNAs (Ren et al., 2014b; Tu et al., 2015; Wang et al., 2015). Although HESO1 and URT1 share many similarities in uridylation, there are also some differences. For miRNA, HESO1 has been proven to be the major enzyme responsible for uridylation of unmethylated miRNAs and siRNAs, while URT1 mainly uridylates miRNAs sequentially and cooperatively with HESO1 (Tu et al., 2015; Wang et al., 2015). For mRNA, URT1 becomes the main TUTase to cover 70%–80% of mRNAs uridylation; however, HESO1 targets mostly the mRNAs with short tails (Sement et al., 2013). HESO1 and URT1 also exhibit distinct substrate preferences, especially the 3′ end nucleotide of its substrate sRNAs, in detail, HESO1 has the strongest preference for U, while URT1 for A (Tu et al., 2015; Wang et al., 2015).

### Exoribonuclease Mediated MicroRNA Degradation

RNA degradation occurs either from its 5′ or 3′ terminal guide by exoribonucleases or cleavage by endoribonuclease theoretically. Different from mRNA, miRNA primarily includes 3′ to 5′ degradation, according to methylation mediated by HEN1. Small RNA-degrading nucleases (SDNs), a family of DEDDh 3′ to 5′ exonucleases, showed nuclease activity by removing 3′ end methylated nucleotides directly and promoted the degradation of some methylated miRNAs (Ramachandran and Chen, 2008; Yu et al., 2017; Figure 2C). SDN1 is responsible for the degradation of single-stranded small RNAs, which is 17–24 nt long. In addition, it is sensitive to methylated miRNAs but not to the miRNA/miRNA* duplex and long single-stranded RNAs (Ramachandran and Chen, 2008; Yu et al., 2017; Chen et al., 2018). Atrimmer 2 (ATRM2), a DEDDy type exoribonucleases, is involved in the degradation of unmethylated miRNA/miRNA* duplexes during RISC assembly. The loss function of ATRM2 in *hen1* mutant partially rescues the developmental defects and the abundance of numerous miRNAs but decreases the expression of the corresponding miRNA targets (Wang X. et al., 2018; Figure 2C).

### Argonaute Protein Mediated MicroRNA Stability Control

AGO protein not only serves as an effector, but also plays critical roles in maintaining the stability of miRNA (Figure 2C). As previously mentioned, several degradative factors, including HESO1, URT1, SDN1, and ATRM2, interact with AGO1 and play an important role in AGO1-bound miRNAs or unmethylated miRNA/miRNA* duplexes. Therefore, AGO1 protein can recruit these regulative factors for the degradation of AGO1-associated miRNAs or unmethylated miRNA/miRNA* duplexes (Ramachandran and Chen, 2008; Chen et al., 2018; Wang X. et al., 2018).

The *Arabidopsis* genome encodes 10 AGO proteins, and it can be grouped into three clades: AGO1/5/10, AGO2/3/7, and AGO4/6/8/9 (Vaucheret, 2008). Different AGO proteins had different functions on their bound miRNAs. AGO1 binds most miRNAs, and the 5′ end of these miRNAs tends to be uridine (Mi et al., 2008). In *ago1* mutant, the abundance of miRNAs at a low level suggests that AGO1 can also stabilize miRNA (Vaucheret et al., 2004). AGO7 and AGO10 are primarily associated with miR390 and miR165/166, respectively. In *ago7* mutant, the expression level of miR390 is high, but the cause remains unknown (Li et al., 2017a). Over-accumulation of miR165/166 in *ago10* mutant is suppressed when AGO10 is overexpressed (Yu et al., 2017). In *Oryza sativa*s, low abundant of miRNAs and phased secondary sRNAs in AGO18 loss function mutants suggested that AGO18 can also stabilize sRNAs including miRNAs (Das et al., 2020). In addition AGO18 can sequester miR168 and prevent it from repressing its target AGO1 essential for antiviral RNAi (Wu et al., 2015). Likely, AGO18 can sequesters miR528 apart from AGO1 to prevent the formation of an effective RISC, as a result, the function of miR528 targeted L-ascorbate oxidase (AO) is then released, following the initiation of ROS-mediated resistance against Rice stripe Tenuivirus (RSV) infection (Wu et al., 2017). In brief, AGO proteins exhibit different functions in miRNA stability control. On the one hand, it protects miRNAs from degradation. On the other hand, it can recruit some degradative factors facilitating the degradation of AGO-bound miRNAs.

### Targets Mediated MicroRNA Stability Control

miRNAs are associated with AGO proteins to direct post-transcriptional target gene repression by the manner of miRNA-target complementation. miRNA-target complementation can also trigger destabilization of miRNAs, for example, target mimicry, which is initially discovered in plants. Phosphate Starvation1 (IPS1), a non-coding RNA, whose sequence provides a complementary motif of miR399, due to its loosely complementary around cleavage site, as a result, miR399 is sequestered from its endogenous targets by IPS1 binding. Consequently, miR399 is sequestered from its endogenous targets (Franco-Zorrilla et al., 2007). Based on the abovementioned principles, some researchers designed target mimicry technologies artificially, such as MIM and Short Tandem Target Mimic (STTM), which can be used to reduce endogenous miRNA function (Todesco et al., 2010; Yan et al., 2012). In 2018, these two research groups found that the loss function of an F-box gene Hawaiian Skirt (HWS) leads to the recovery of MIM and STTM-induced developmental and molecular defects (Lang et al., 2018; Mei et al., 2019). In *hws* mutant, miRNA and its mimicry targets coexisted with AGO1 stably, showing that HWS may touch off unoptimizable RISCs degradation (Mei et al., 2019; Figure 2C). RISC interacts with Clearing 3′–5′ exoribonucleases 1/2 (RICE 1/2), which are two members of DnaQ-like exonucleases. Malfunction of RICE1 and RICE2 resulted in reduced miRNA levels. RICE1 and RICE2 interact with AGO1 and AGO10, thereby degrading uridylated 5′ products of miRNA cleavage products to maintain the normal function of RISC (Ren et al., 2014a; Zhang et al., 2017b; Zuber et al., 2018; Figure 2C). Therefore, the stability of RISC-associated miRNAs is impaired by the excessive accumulation of cleaved products.

## Future Perspectives

The transcription, stability, processing of pri-miRNA, miRNA loading, and stability control require multiple proteins and their cooperation to maintain normal function. In recent years, a large number of factors involved in miRNA biogenesis and stability control have been identified, and the molecular mechanism has been revealed. However, many questions still persist. For example, pri-miRNA processing occurs in D-bodies; however, the formation of D-bodies and how the core members of the processing machinery located at D-bodies remain unknown. DCL1 plays a critical role in processing, but little is known about the post-translational modification of DCL1. Moreover, the regulation of DCL1 activity and HEN1 remains unknown. 3′-end modification, AGOs, and targets also affect the miRNA stability, but the detailed mechanisms remain unknown. Apart from terminal modification, other modifications (N^6^-methyladenosine, 5-methylcytidine, N^1^-methyladenosine, pseudouridine, and so on) for pri-miRNAs and/or miRNAs and their biological function remain to be further explored. Further studies should identify and characterize more factors to clarify the biological function and significance of these factors in all of these processes, particularly in miRNA stability control.

## Author Contributions

LZ and SG drafted the manuscript and revised it. YX, MS, and XJ retrieved and collected the references of miRNA biogenesis and drafted the first picture. SC and ZH retrieved and collected the other references and drafted the second picture. All authors have read and agreed to the publishment of this version.

## Conflict of Interest

The authors declare that the research was conducted in the absence of any commercial or financial relationships that could be construed as a potential conflict of interest.

## Publisher’s Note

All claims expressed in this article are solely those of the authors and do not necessarily represent those of their affiliated organizations, or those of the publisher, the editors and the reviewers. Any product that may be evaluated in this article, or claim that may be made by its manufacturer, is not guaranteed or endorsed by the publisher.
